# Effects of Interactions of Auxin-Producing Bacteria and Bacterial-Feeding Nematodes on Regulation of Peanut Growths

**DOI:** 10.1371/journal.pone.0124361

**Published:** 2015-04-13

**Authors:** Li Xu, Wensi Xu, Ying Jiang, Feng Hu, Huixin Li

**Affiliations:** Soil Ecology Lab, College of Resources and Environmental Sciences, Nanjing Agricultural University, Nanjing, Jiangsu, P.R. China; Chinese Academy of Sciences, CHINA

## Abstract

The influences of an IAA (indole-3-acetic acid)-producing bacterium (*Bacillus megaterium*) and two bacterial-feeding nematodes (*Cephalobus* sp. or *Mesorhabditis* sp.) on the growth of peanut (*Arachis hypogaea* L. cv. Haihua 1) after various durations of time were investigated in natural soils. The addition of bacteria and nematodes and incubation time all significantly affected plant growth, plant root growth, plant nutrient concentrations, soil nutrient concentrations, soil microorganisms and soil auxin concentration. The addition of nematodes caused greater increases in these indices than those of bacteria, while the addition of the combination of bacteria and nematodes caused further increases. After 42-day growth, the increases in soil respiration differed between the additions of two kinds of nematodes because of differences in their life strategies. The effects of the bacteria and nematodes on the nutrient and hormone concentrations were responsible for the increases in plant growth. These results indicate the potential for promoting plant growth via the addition of nematodes and bacteria to soil.

## Introduction

Soil bacteria significantly affect plant growth. These bacteria can directly support plant growth through the mineralisation of nutrients, such as nitrogen (N) and phosphorus (P), in terrestrial ecosystems. They can also indirectly stimulate plant growth or prevent the deleterious effects of environmental stresses via the microbial production of chemical compounds, such as phytohormones, including auxins, cytokinins, and antibiotics [1–3].

Soil nematodes, which share an environment with bacteria, are critical participants in fundamental ecological processes that occur in soils, including mineralisation and nutrient cycling [4,5]. Certain nematodes may harbour specific symbiotic bacterial communities, and they can influence the abundance and composition of bacterial taxa. The nematodes can even provide protection to bacteria and disperse them to more favourable environments [6–8]. Their selectivity has been attributed to variations in odours released by bacteria [9] and to physical characteristics, such as variations in bacterial size and shape [10]. There are even examples of some closely related nematode species that prefer different bacterial strains [11].

The grazing of bacterial-feeding nematodes on bacteria can accelerate bacterial colonisation and microbial activity, which can promote plant growth [12]. One potential mechanism of the bacterial promotion of plant growth involves an increase in the release of nutrients readily available to plants, and this increase may be attributed to an increase in bacterial turnover caused by nematode grazing [13] or to the excretion of ingested microbial biomass N by nematodes [14]. Furthermore, nematode grazing may alter the microbial community structure, stimulating the activity of certain plant growth-promoting rhizobacteria (PGPR), such as auxin-producing bacteria, which can promote plant growth via the release of hormones [15,16]. However, the selectivity and flexibility of free-living nematodes with regard to feeding are generally poorly understood [17], and the effects of various nematode species on soil microflora, and even on plant growth, likely vary [18]. Therefore, the precise mechanisms underlying the effects of the interactions of nematodes and bacteria on plant growth are still not fully understood. The addition of indole-3-acetic acid (IAA)-producing bacteria may directly improve plant growth and induce pathogen resistance [19]. Whether PGPR are influenced by nematode grazing and ultimately affect plant growth require further investigation.

In this study, we evaluated two types of bacterial-feeding nematodes from different coloniser-persister (cp) scale groups and thus different life strategies[20], applied individually or in combination with a type of IAA-producing bacteria, to determine the effects on peanut growth. In addition, we aimed to determine the mechanisms underlying the effects of the interactions of these nematodes and this bacterium on nutrient and hormone concentrations in soil and further the influence on the peanut plants. In contrast with previous studies, which were generally performed under gnotobiotic microcosm conditions, natural soil was used as the growth substrate in this study. Hence, the results may be valid for future agricultural applications.

## Materials and Methods

### 1. Soils

Sandy loam alluvial soil (57.5% sand, 26.6% silt and 15.9% clay) was collected from Banqiao, Nanjing City, Jiangsu Province, People’s Republic of China. No specific permits were required for the described field studies. The location of collection was not privately owned or protected, and the field studies did not involve endangered or protected species. Fresh soil was sieved through a 5 mm mesh to remove stones, macrofauna and discernible plant material. The soil contained 10.87μg g^-1^ organic C, 0.89μg g^-1^ total N, 2.15μg g^-1^ NH_4_^+^-N and 28.03μg g^-1^ NO_3_^-^-N, with a pH (H_2_O) of 6.43.

### 2. Bacteria

The test bacteria, *Bacillus megaterium* JX 15, was isolated from the experimental soil and cultured in the laboratory. After 24 h of incubation, the bacteria produced high levels of IAA, which peaked at 22.55μg L^−1^.

The bacteria were cultured in Luria Bertani (LB) broth for 48 h at 28°C at a shaking speed of 150 rev. min^-1^. Then, the cultures were centrifuged (5000 *g*, 5 min). The pellets were washed twice with sterile water and then resuspended in sterile water to inoculate the soil at 10^6^ CFU g^-1^ dry soil.

### 3. Nematodes

Two bacterial-feeding nematodes, a *Cephalobus* sp. and a *Mesorhabditis* sp. that were isolated from the experimental soil and cultured in the laboratory, were evaluated.

The nematodes were cultured in Petri dishes containing nematode growth medium (NGM) at 22°C for 10 d and fed *B*. *megaterium* JX 15. When required, at 2 d after the addition of bacteria, nematodes were collected from dishs by rinsing with sterile water, after which they were concentrated by centrifugation, washed five times with sterile distilled water, and added to the soil at a rate of 30 ind. g^-1^ dry soil.

### 4. Plants

Seeds of peanut (*Arachis hypogaea* L. cv. Haihua 1) were surface sterilised with 20% H_2_O_2_ for 20 min. After sterilisation, the seeds were rinsed five times with sterile distilled water and dried on sterile filter disks. Several seeds were pre-germinated on moist filter paper in the dark for 2 d at 20°C to synchronise germination.

### 5. Experimental design and incubation conditions

Ninety grams of soil were weighed and placed into each pot. Six different treatments were included as follows: (1) control soil without bacteria or nematodes (CK); (2) soil inoculated with *B*. *megaterium* JX 15 (B); (3) soil inoculated with the isolated *Cephalobus* sp. (N1); (4) soil inoculated with the isolated *Mesorhabditis* sp. (N2); (5) soil inoculated with *B*. *megaterium* JX 15 and the *Cephalobus* sp. (BN1); and (6) soil inoculated with *B*. *megaterium* JX 15 and the *Mesorhabditis* sp. (BN2). and each treatment had 9 replicates.

After inoculation of both the bacteria and nematodes, two germinated peanuts were planted in each pot. The pots were maintained in a temperature/light-controlled incubation room (22°C/18 h day length). Distilled water was evenly applied to the soil surface to maintain the moisture content at 24.8% (w/w) for the duration of the experiment. At 14, 28, and 42 d of growth, three replicates were sampled for each treatment for plant and soil analyses.

### 6. Analytical methods and statistical analysis

At the end of the experiment, the plants were harvested and separated into foliage and root portions. The height and dry weight of the foliage were measured. To determine the macronutrient concentrations (i.e., N, P and K), plants were oven-dried and ground and then digested by the H_2_SO_4_-H_2_O_2_ method. A continuous-flow autoanalyser (Seal Auto Analyser 3, Germany) was used to determine the plant total N concentration, and the plant total P concentration was determined by the molybdenum blue colorimetric method. The plant K concentration was determined by flame emission spectrometry [21].

The roots were scanned using an LA1600^+^ scanner (Regent Instruments Inc., Quebec, Canada), and the total root length, mean diameter, total surface area, and number of tips were analysed using Win-rhizo software (Win-rhizo 2003b; Regent Instruments Inc., Quebec City, Quebec, Canada).

Nematodes were extracted from 10 g of fresh soil according to a modification of the Baermann method, using trays instead of funnels [22]. After 48 h of extraction at room temperature, the nematode suspension was collected. The number of nematodes in the suspension was determined using a dissecting microscope.

Soil NH_4_^+^-N and NO_3_^-^-N were extracted with a 1 mol L^-1^ KCl solution (soil:KCl 1:5) for 30 min, and the concentrations were determined using a continuous-flow autoanalyser (Seal Auto Analyser 3, Germany). The concentrations are expressed on a dry weight basis. The available soil P was determined using the Olsen P method based on the extraction of air-dried soil with 0.5 M NaHCO_3_, pH 8.5. Microbial biomass C was determined by the chloroform fumigation-direct extraction method [12]. The soil IAA concentration was measured by high-performance liquid chromatography (HPLC) [23]. Soil basal respiration (BR) was measured by the alkali (1 M NaOH) absorption of CO_2_ produced over 24 h, followed by the titration of residual OH^-^ using a standardised acid [12].

All of the obtained data were subjected to statistical analyses using SPSS 16.0 software for Windows. Three-way ANOVAs were performed to determine whether the effects of the addition of the IAA-producing bacteria or bacterial-feeding nematodes or those of incubation time on plant growth, the plant nutrient concentrations, the soil IAA concentration, the soil nutrient concentrations or the soil microorganisms were significant. One-way ANOVAs were performed to determine the significances of the differences among the treatments for each time point individually. Significant differences among means were determined with Duncan’s multiple-range tests (*P* < 0.05).

## Results

### 1. Dynamics of nematodes in soil

For the CK treatment, a consistent number of approximately 10 ind. g^-1^ dry soil of nematodes was observed regardless of the incubation time. For the other treatments, the numbers of nematodes varied over time, generally peaking at 28 d, before subsequently decreasing by day 42 (P < 0.05, Table 1).

**Table 1:** Dynamics of bacterial-feeding nematodes in soil.

Samplingtime	Number of nematodes (ind. g^-1^ dry soil)					
	CK	B	N1	N2	BN1	BN2
**14**	13.58±2.31Ac	18.33±2.93Babc	20.28±3.49Bab	16.32±3.34Bbc	22.19±2.04Ba	21.96±2.96Ba
**28**	10.46±2.57Ad	24.97±2.80Ac	28.90±2.80Abc	26.89±2.51Ac	32.97±2.12Aab	36.03±2.63Aa
**42**	11.25±1.64Ab	9.47±1.33Cc	19.11±1.99Ba	17.33±2.14Ba	20.06±2.02Ba	19.87±1.50Ba

The values are the mean±standard deviation (SD) (n = 3), and those with the same letter in each row or column indicate the absence of a significant difference among the different treatments and different days (P > 0.05). The capital letters indicate significant differences among the different days, and the lowercase letters represent significant differences among the different treatments. CK: control soil without bacteria or nematodes; B: soil inoculated with *B*. *megaterium* JX 15; N1: soil inoculated with the isolated *Cephalobus* sp.; N2: soil inoculated with the isolated *Mesorhabditis* sp.; BN1: Soil inoculated with *B*. *megaterium* JX 15 and the *Cephalobus* sp.; and BN2: soil inoculated with *B*. *megaterium* JX 15 and the *Mesorhabditis* sp.

The nematode abundance was increased in the B treatments compared with the CK treatment, except after 42 d of incubation. The N1 and N2 treatments accelerated the increase in nematodes, but this increase was only significant after 42 d of incubation. The BN1 and BN2 treatments resulted in the highest nematode abundances, while no significant differences were observed between the two nematodes.

### 2. Changes in plant growth, plant nutrient concentrations and root architecture

The interaction between the bacteria and bacterial-feeding nematodes significantly enhanced plant growth (Table 2). When combined with the addition of bacteria, the effects of the two nematode treatments on increasing plant height and shoot dry weights differed after 42 d. At this time point, the plant height and shoot dry weights were significantly greater for the BN2 treatment compared with the BN1 treatment (P< 0.05). The plant nutrient concentrations were increased by the addition of either bacteria or nematodes in the B, N1, and N2 treatments (P< 0.05) and were further increased following the combined addition of bacteria and nematodes in the BN1 and BN2 treatments (P< 0.05). No significant differences were observed between the two nematode treatments (P> 0.05).

**Table 2:** Effects of various bacteria and nematode treatments on peanut plant.

Samplingtime	Treatment	Plant Height (cm)	Shoot Dry Weight (g plant^-1^)	N (%)	P (%)	K (%)
**14d**	**CK**	4.32±0.21 d	0.48±0.02 b	1.19±0.02 c	0.011±0.002 b	0.31±0.01 b
	**B**	6.33±0.36 c	0.51±0.04 b	1.31±0.01 b	0.018±0.002 a	0.48±0.01 a
	**N1**	7.79±0.35 b	0.72±0.05 a	1.34±0.01b	0.019±0.002 a	0.50±0.03a
	**N2**	7.53±0.61 b	0.72±0.05 a	1.39±0.02 b	0.018±0.002 a	0.54±0.02 a
	**BN1**	9.94±0.43a	0.75±0.03 a	1.66±0.08 a	0.020±0.004 a	0.57±0,04a
	**BN2**	10.54±0.55a	0.77±0.01 a	1.69±0.04a	0.022±0.002 a	0.60±0.03a
**28d**	**CK**	8.54±0.15 d	0.62±0.01 c	1.37±0.06 c	0.019±0.003 d	0.43±0.03 c
	**B**	9.93±0.57 c	0.75±0.04 b	1.63±0.05 b	0.029±0.002 c	0.77±0.02 b
	**N1**	10.02±0.7 c	0.77±0.10 b	1.65±0.05 b	0.043±0.004 b	0.82±0.02 a
	**N2**	11.97±0.19b	0.85±0.08 b	1.71±0.06 b	0.046±0.002 b	0.85±0.04 a
	**BN1**	12.20±0.31 b	1.04±0.06 a	2.46±0.12 a	0.056±0.004 a	0.93±0.003a
	**BN2**	13.65±0.20 a	1.01±0.02 a	2.49±0.03 a	0.053±0.001 a	1.02±0.002a
**42d**	**CK**	13.50±0.25 d	0.66±0.04 d	1.61±0.03d	0.027±0.003 d	0.56±0.03 c
	**B**	15.60±0.51c	0.80±0.02 c	2.19±0.03c	0.038±0.005 c	1.08±0.02 b
	**N1**	15.97±0.43 c	0.81±0.06 c	2.33±0.06bc	0.051±0.002b	1.11±0.03 b
	**N2**	15.93±0.36 c	0.87±0.04 c	2.47±0.02b	0.053±0.004 b	1.13±0.02 b
	**BN1**	17.51±0.38 b	1.04±0.02 b	4.13±0.06 a	0.066±0.004 a	1.23±0.05 a
	**BN2**	19.64±0.43 a	0.19±0.08 a	4.37±0.07 a	0.073±0.002 a	1.21±0.02 a

Note: The values (mean±SD, n = 3) within rows that are followed by different letters represent significant differences (P< 0.05) at each sampling time. CK: control soil without bacteria or nematodes; B: soil inoculated with *B*. *megaterium* JX 15; N1: soil inoculated with the isolated *Cephalobus* sp.; N2: soil inoculated with the isolated *Mesorhabditis* sp.; BN1: soil inoculated with *B*. *megaterium* JX 15 and the *Cephalobus* sp.; and BN2: soil inoculated with *B*. *megaterium* JX 15 and the *Mesorhabditis* sp.

**Fig 1:**
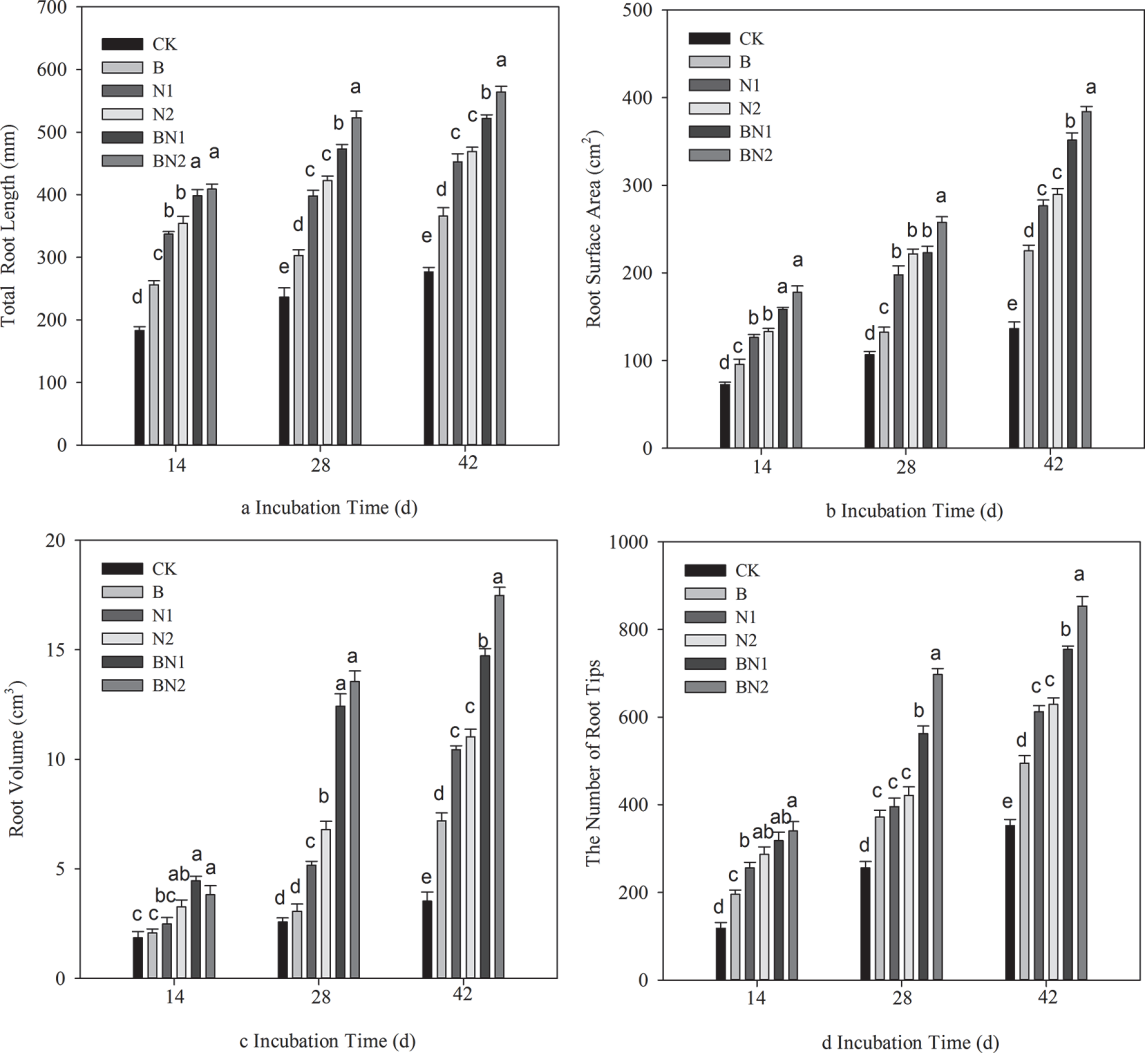
Effects of various bacteria and nematode treatments on the root architecture of peanut: (a) total root length; (b) root surface area; (c) root volume; and (d) the number of root tips. The error bars represent the standard deviation. For each incubation time, the different letters indicate significant differences (*P* < 0.05). CK: control soil without bacteria or nematodes; B: soil inoculated with *B*. *megaterium* JX 15; N1: soil inoculated with the isolated *Cephalobus* sp.; N2: soil inoculated with the isolated *Mesorhabditis* sp.; BN1: soil inoculated with *B*. *megaterium* JX 15 and the *Cephalobus* sp.; and BN2: soil inoculated with *B*. *megaterium* JX 15 and the *Mesorhabditis* sp.

The root length, surface area, volume and number of root tips were significantly increased with the duration of cultivation (Fig 1). Significantly greater increases in root growth were observed following the N1 and N2 treatments compared with the B treatment, and it was further promoted by the BN1 and BN2 treatments. The addition of the different nematodes did not significantly change the root length; however, the addition of the combination of *B*. *megaterium* and the *Mesorhabditis* sp. (BN2) resulted in significantly greater increases in root length compared with those resulting from the addition of the *Cephalobus* sp. (BN1) on days 28 and 42. The addition of bacteria and nematodes, both individually and in combination, increased the root surface area, root volume and number of root tips compared with the control treatment. Notably, on day 42, similar to what was observed for the root length, the addition of the combination of *B*. *megaterium* and the *Mesorhabditis* sp. (BN2) caused significantly greater increases in the aforementioned root characteristics compared with the addition of the *Cephalobus* sp. (BN1).

According to three-way ANOVAs, the addition of bacteria and nematodes and incubation time both significantly affected plant height, plant shoot weight, plant root growth, and plant nutrient concentrations, and the effects of the bacteria × time and nematode × time interactions on these indices were also significant (Table 3, P<0.05). However, the bacteria × nematode interaction did not significantly affect root length or plant P concentration. The bacteria × nematode × time interaction had significant effects on plant growth, plant root growth, and plant nutrient concentrations but not on root area (Table 3, P>0.05).

**Table 3:** F-values and mean squared errors for three-way ANOVAs analysing the effects of bacteria (*B*. *megaterium* JX 15, B) and nematodes (with or without *Cephalobus* sp. or *Mesorhabditis* sp., N) on plant growth, plant root growth, and plant nutrient concentrations in addition to incubation time (T).

	d.f.	Plant Growth		Plant Root Growth				Plant Nutrient Concentrations		
		Plant Height	Shoot Weight	Root Length	RootArea	Root Volume	Root Tips	Plant N	Plant P	Plant K
**B**	1	334.22	73.89	907.54	858.31	1.44E3	1.03E3	854.60	58.78	473.20
**N**	2	302.71	127.63	2.14E3	1.77E3	1.56E3	1.10E3	549.80	134.33	486.66
**T**	2	1.75E3	89.64	745.27	2.65E3	2.32E3	2.31E3	1.32E3	296.33	1.52E3
**B×N**	2	6.41	9.10	2.87^NS^	7.07	122.43	26.34	14.24	1.44^NS^	96.08
**B×T**	2	3.46	12.80	6.75	119.20	196.34	75.67	53.65	10.78	26.62
**N×T**	4	7.35	5.84	8.34	85.07	211.62	45.48	34.87	19.67	23.77
**B×N×T**	4	3.43	6.67	3.57	1.46^NS^	49.42	13.02	26.00	3.44	15.81
**Mean Squared Error**	36	0.00	0.02	86.01	38.45	0.12	258.63	0.00	1.67E-5	0.00

The test was significant at the 5% level (P < 0.05).

NS: the test was not significant at the 5% level.

### 3. Soil NH_4_^+^-N, NO_3_^-^-N, mineral N and available P

**Fig 2:**
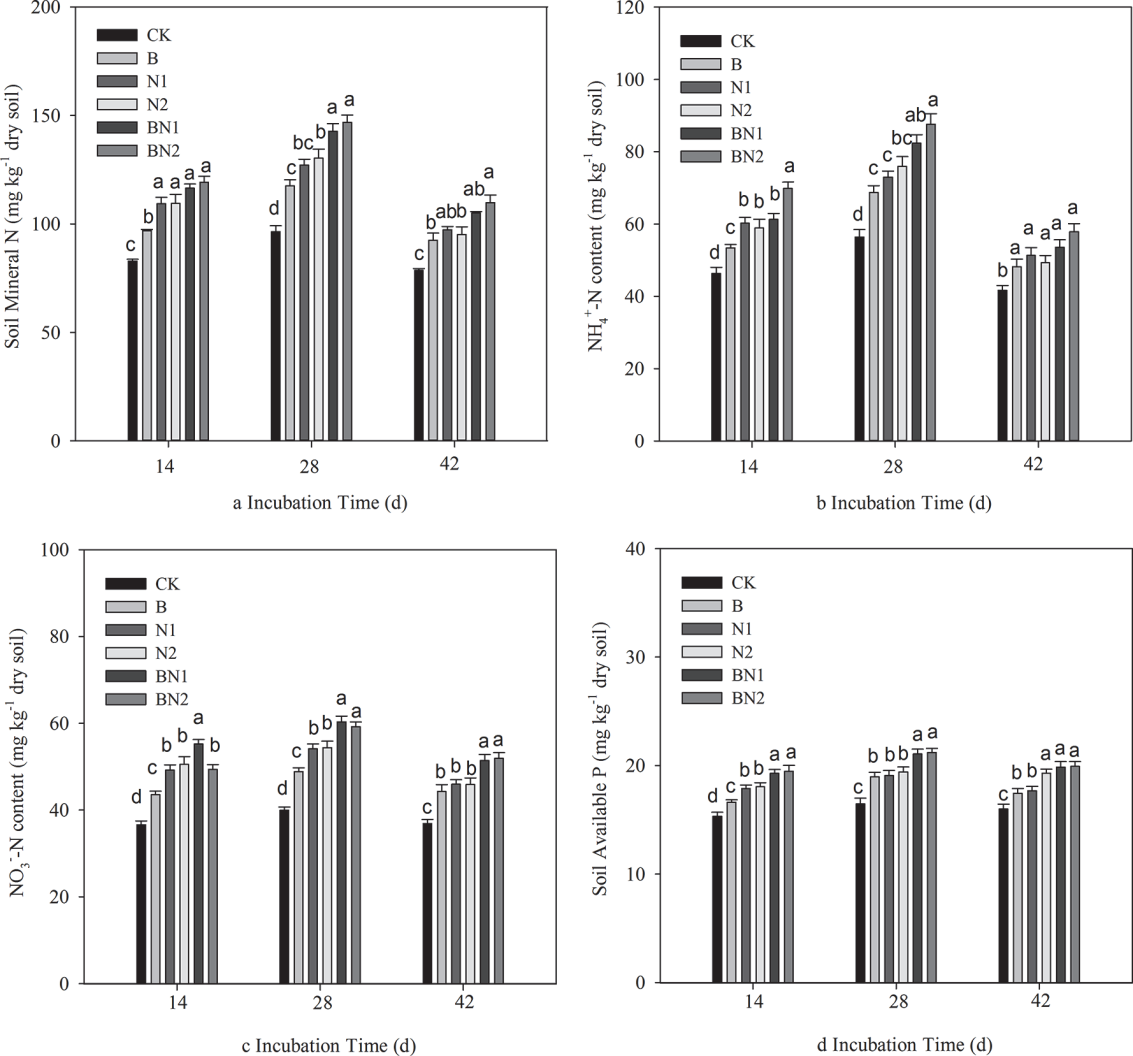
Effects of bacterial-feeding nematodes on soil mineral N concentration and available soil P. a: soil mineral N; b: NH_4_^+^-N concentration; c: NO_3_^-^-N concentration; and d: soil available P. The error bars represent the standard deviation. For each incubation time, the different letters indicate significant differences (*P* < 0.05). CK: control soil without bacteria or nematodes; B: soil inoculated with *B*. *megaterium* JX 15; N1: soil inoculated with the isolated *Cephalobus* sp.; N2: soil inoculated with the isolated *Mesorhabditis* sp.; BN1: soil inoculated with *B*. *megaterium* JX 15 and the *Cephalobus* sp.; and BN2: soil inoculated with *B*. *megaterium* JX 15 and the *Mesorhabditis* sp.

During incubation, the concentrations of soil NH_4_^+^-N, NO_3_^-^-N, mineral N and available P increased until day 28, after which they subsequently decreased by day 42 (Fig 2), and significant effects were observed following the addition of bacteria or/and nematodes.

The addition of bacteria and nematodes increased the soil mineral N concentration compared with the control treatment (CK). This increase was greater for the N1 and N2 treatments compared with the B treatment. No significant differences were observed between the N1 and N2 treatments. The combination of bacteria and nematodes resulted in the highest soil mineral N concentrations, particularly on days 28 and 42. No significant differences were observed between BN1 and BN2. Similar results were also observed for the soil NH_4_^+^-N, NO_3_^-^-N, and available P concentrations.

According to three-way ANOVAs, the addition of bacteria and nematodes and incubation time all significantly affected the soil nutrient concentrations. The effects of the bacteria × time interaction on these indices were also significant (Table 4, P<0.05). However, the soil P concentration was not significantly affected by the bacteria × nematode interaction, the nematode × time interaction or the bacteria × nematode × time interaction (Table 4, P>0.05). In addition, the bacteria × nematode × time interaction only had a significant effect on soil NO_3_^-^-N.

**Table 4:** F-values and mean squared errors for three-way ANOVAs analysing the effects of bacteria (*B*. *megaterium* JX 15, B) and nematodes (with or without *Cephalobus* sp. or *Mesorhabditis* sp., N) on soil nutrient concentrations in addition to incubation time (T).

	d.f.	Soil Nutrient Concentrations			
		Soil Mineral N	Soil NH_4_^+^-N	Soil NO_3_^-^-N	SoilP
**B**	1	308.21	197.71	290.36	200.07
**N**	2	415.83	242.83	460.47	221.16
**T**	2	556.94	635.41	155.77	64.56
**B×N**	2	5.62	11.05	15.48	2.32^NS^
**B×T**	2	8.71	9.32	6.60	3.99
**N×T**	4	11.90	10.93	8.41	1.36^NS^
**B×N×T**	4	0.60^NS^	1.32^NS^	4.82	1.91^NS^
**Mean Squared Error**	36	7.84	4.11	1.47	0.18

The test was significant at the 5% level (P < 0.05).

NS: the test was not significant at the 5% level.

### 4. Microbial biomass C, soil respiration and soil IAA concentrations

**Fig 3:**
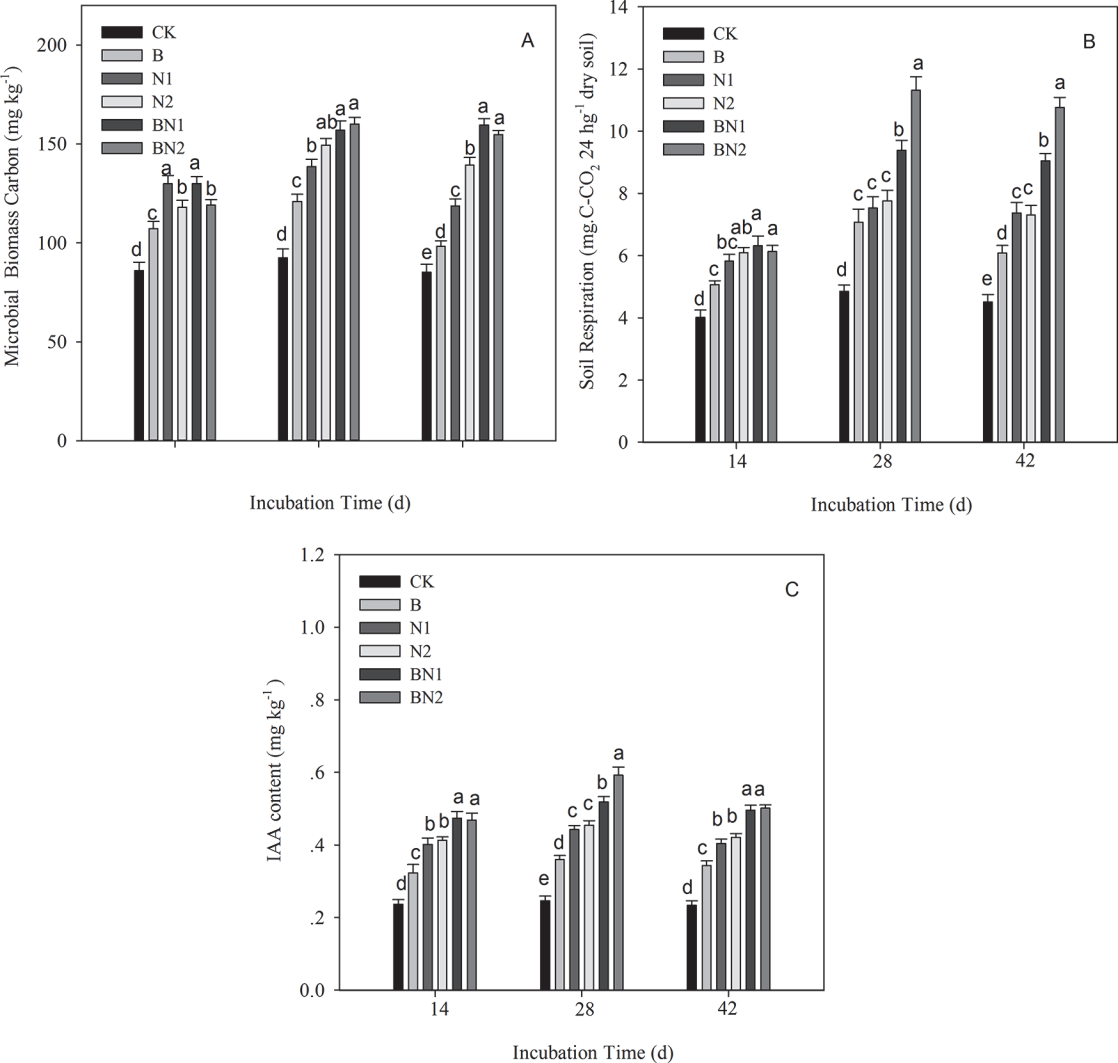
Dynamics of soil microbial biomass C (A), soil respiration (B), and IAA concentration (C) under various bacteria and nematode treatments. **The error bars represent standard deviations.** The different letters indicate significant differences for each incubation time (*P* < 0.05). CK: control soil without bacteria or nematodes; B: soil inoculated with *B*. *megaterium* JX 15; N1: soil inoculated with the isolated *Cephalobus* sp.; N2: soil inoculated with the isolated *Mesorhabditis* sp.; BN1: soil inoculated with *B*. *megaterium* JX 15 and the *Cephalobus* sp.; and BN2: soil inoculated with *B*. *megaterium* JX 15 and the *Mesorhabditis* sp.

The microbial biomass C and soil respiration were increased significantly by 28 d of peanut growth, and their levels remained stable until 42 d of growth following the various bacteria and nematode treatments compared with the control treatment. The soil IAA concentration was also increased following 28 d of incubation, but the level subsequently decreased by day 42 (Fig 3).

The microbial biomass C increased under the bacteria or/and nematode treatments compared with the control treatment, and the addition of nematodes in the N1 and N2 treatments caused a greater increase compared with that of the bacteria in the B treatment (Fig 3A). With regard to the two nematode treatments (N1 and N2), microbial biomass C was significantly greater following the addition of the *Cephalobus* sp. (N1) compared with that of the *Mesorhabditis* sp. (N2) on day 14 of treatment. However, with increasing incubation time, the *Mesorhabditis* sp. in the N2 treatment caused a significantly greater increase in microbial biomass C compared with the *Cephalobus* sp. in the N1 treatment, particularly on day 42. With regard to the two bacteria and nematode combination treatments (BN1 and BN2), BN1 caused a greater increase in microbial biomass C than BN2 on day 14, but with increasing incubation time, this difference was no longer significant.

Soil respiration was also increased by the addition of bacteria or/and either nematode compared with the CK treatment (Fig 3B). The addition of the two nematodes caused similar increases in soil respiration, and when combined with bacteria, the *Mesorhabditis* sp. (BN2) caused a significantly greater increase than the *Cephalobus* sp. (BN1).

The introduction of bacteria or nematodes significantly increased the soil IAA concentration compared with the control treatment (Fig 3C), and the combination of bacteria and nematodes resulted in the highest IAA concentration. There were no significant differences between the two nematode treatments, with the exception of the IAA concentration, which was significantly greater following the BN2 treatment compared with the BN1 treatment on day 28 of peanut growth.

According to three-way ANOVAs, the bacteria and nematode additions and the incubation time all significantly affected the soil microorganism and soil IAA concentrations. All of these indices were significantly affected by the bacteria x nematode x time interaction (Table 5, P<0.05), except for the soil IAA concentration, which was not significantly affected by the bacteria × nematode interaction (Table 5, P>0.05).

**Table 5:** F-values and mean squared errors for three-way ANOVAs analysing the effects of bacteria (*B*. *megaterium* JX 15, B) and nematodes (with or without *Cephalobus* sp. or *Mesorhabditis* sp., N) on soil microorganism characteristics and soil IAA concentration in addition to incubation time (T).

	d.f.	Soil Microorganism		Soil Auxin Concentration
		Soil MBC	Soil Respiration	Soil IAA Concentration
**B**	1	277.87	499.03	515.83
**N**	2	758.71	515.72	845.98
**T**	2	152.87	346.72	53.83
**B×N**	2	14.49	14.37	2.72^NS^
**B×T**	2	22.26	62.90	7.31
**N×T**	4	35.32	26.57	7.05
**B×N×T**	4	18.46	12.13	3.53
**Mean Squared Error**	36	13.38	0.09	0.00

The test was significant at the 5% level (P < 0.05).

NS: the test was not significant at the 5% level.

## Discussion

There is widespread evidence that soil fauna interact with microorganisms and play important roles in regulating soil ecosystem processes and plant growth. Among the soil fauna, bacterial-feeding nematodes, which are the most abundant metazoans in soil, have been suggested to stimulate soil N mineralisation and promote plant growth by grazing on microorganisms, consequently releasing nutrients from the consumed microbial biomass [24]. Furthermore, a number of studies have reported that nematodes and protozoa can stimulate plant growth via hormonal effects, significantly influencing the early development of plant roots and subsequently improving plant growth [15,25]. However, these effects vary among nematode species, microbial species, and plant species and also differ depending on the C/N ratios of substrates [26], and the mechanisms underlying the effects of nematodes are not fully understood. In this study, the effects of the interaction between a bacterium and two bacterial-feeding nematodes on plant growth were evaluated in a natural soil system rather than a gnotobiotic system, thereby increasing the relevance of these results to field management practices [12, 27].

Our results demonstrated that the introduction of bacteria or/and nematodes increased the number of nematodes in the soil, particularly after 28 d of peanut growth (Table 1). This finding may be attributed to the increased amount of microbial biomass that was available as a food source for the nematodes (Fig 3). After 42 d, the numbers of nematodes declined, which may have been due to competition for food resources, considering that the microbial biomass was slightly decreased. Although the two types of bacterial-feeding nematodes differ based on their life strategies; i.e. the *Mesorhabditis* sp. (Rhabditidae, cp-1) is characterised by higher metabolic activity and higher adaptability compared with the *Cephalobus* sp. (cp-2), and the *Mesorhabditis* sp. can drastically increase in density in nutrient-rich environments, while the density of *Cephalobus* sp. may be increased over the long-term and in conditions of limited resources [28,29], the abundance of these soil nematodes did not significantly differ in this experiment (Table 1). This finding may be due to the fact that the two nematodes were isolated from the same soil; therefore, as a result of environmental adaptation, differences between their life strategies may have been diminished. The short incubation time may have also contributed to this result.

The treatments involving the addition of bacteria and bacterial-feeding nematodes resulted in enhanced plant growth and more highly branched root systems with longer and thicker roots (Table 2, Fig 1). These results are in agreement with previous studies of tomatoes and *Arabidopsis thaliana* [12,15]. Two previously described mechanisms (involving the effects of interactions between bacteria and nematodes on nutrient availability and auxin concentration) are also supported by our research results. The addition of the bacteria or/and nematodes significantly increased the concentrations of mineral N and available P in the natural soil (Fig 2); furthermore, the plant nutrient concentrations were also increased (Table 2). Positive effects of the treatments were observed in the increasing order of bacteria and nematode >nematode > bacteria. This order is likely to be attributed to the increased biomass and activities of the soil microorganisms (Fig 3). The grazing activities of the bacterial-feeding nematodes may have stimulated the soil microorganism community e.g. by affecting nitrification and the rate of transformation of soil mineral N [13,30]. In addition, the nematodes may have excreted the ingested microbial biomass, providing N in an available form, thus influencing soil nutrient dynamics [14].

The availability of soil nutrients increased over the first 28 d and subsequently decreased by day 42. This finding might be related to peanut plant growth, which determines the timing of nutrient uptake, and/or to decreases in the numbers of nematodes (Table 1).

In addition to its effects on the nutrient concentrations, the introduction of bacteria or/and nematodes also significantly increased the soil IAA concentration (Fig 3). *B*. *megaterium* that was isolated from the same soil could produce IAA, and its addition improved plant growth. Some studies have demonstrated that *B*. *megaterium* influences plant growth and development by regulating plant hormone levels through auxin- and ethylene-independent signalling mechanisms [31–33]. When this bacterium was added in combination with either nematode, the soil IAA concentration was further increased. Other studies have also reported that bacterial-feeding nematodes can stimulate the activity of indigenous PGPR and increase soil auxin concentrations [16,18]. *Bacillus*, a widely studied bacterial genus, has been reported to increase yield and fruit nutrient concentrations under field conditions and to alleviate stress in many other systems [34,35]. Its underlying mechanisms involve stress tolerance, the solubilisation of P, and the production of IAA and siderophores and even antifungal metabolites [19,36]. And some of these bacteria have nematicidal potentials and can confer protection against root-knot nematode infections [37,38]. *Bacillus* species associated with entomopathogenic nematodes may be used as biocontrol agents against postharvest fungal disease [39]. Therefore, in addition to the plant growth-promoting effects of *Bacillus*, its interactions with nematodes require further research.

Three-way ANOVA revealed that the effects of the bacteria, nematodes, and incubation time as well as their interactions all significantly influenced plant growth, plant nutrient concentrations, soil nutrient concentrations and soil IAA concentration (Table 3). On day 42 of cultivation, plant growth was found to be significantly correlated with the soil IAA and soil nutrient concentrations (*P* < 0.05, data not shown). Therefore, the increases in plant and plant root growth observed in the natural soils amended with the bacteria and nematodes were attributed to the effects of their interactions on the soil nutrient and auxin concentrations [12].

A comparison of the two nematodes revealed similar effects on increasing plant growth, except after 42 d, when the plant growth-promoting effects of the *Mesorhabditis* sp. were greater than those of the *Cephalobus* sp., particularly in combination with *B*. *megaterium* (Fig 1). These results may have been due to the different feeding habit of the *Mesorhabditis* sp. (Rhabditidae) compared with the *Cephalobus* sp. (Cephalobidae). For example, the former consumes bacteria non-selectively at high rates, while the latter is a general opportunist that selectively consumes bacteria [28,29]. The addition of these nematodes influenced soil respiration accordingly, with greater respiration occurring in the presence of the *Mesorhabditis* sp. compared with the *Cephalobus* sp. (Fig 3), which may have accelerated the turnover of soil nutrients and facilitated plant growth.

Microbial biomass C did not differ significantly between the two treatments with the different nematodes combined with *B*. *megaterium* after 28 and 42 d of incubation. In addition, most of the plant and soil nutrient concentrations (concentrations of mineral N, NH_4_^+^-N, NO_3_^-^-N, and available P, Fig 2) and the IAA concentration were similar for the two nematode treatments after 42 d of peanut growth. This finding may be attributed to the similar amounts of soil microbial biomass and nematodes in addition to the short incubation time.

In conclusion, the combined addition of nematodes and bacteria can result in significant increases in the concentrations of available nutrients in soil as well as the IAA concentration, promoting plant growth, especially root growth, compared with the individual addition of either nematodes or bacteria. After the longest period of peanut growth (42 d), the effects of the two nematodes on soil respiration differed significantly, likely because of their differing feeding habits. To fully understand the environmental impact and ecological effects of IAA-producing bacteria and bacterial-feeding nematodes, studies of various types of nematodes with longer incubation times should be conducted in the future, and changes in the communities of nematodes and bacteria should also be examined.
